# Mild Hypofractionated Radiation Therapy for Merkel Cell Carcinoma

**DOI:** 10.7759/cureus.6786

**Published:** 2020-01-27

**Authors:** Toshiaki Matsui, Naoko Okano, Hidemasa Kawamura, Takahiro Oike, Tatsuya Ohno

**Affiliations:** 1 Department of Radiation Oncology, Gunma University Graduate School of Medicine, Maebashi, JPN; 2 Heavy Ion Medical Center, Gunma University, Maebashi, JPN

**Keywords:** merkel cell carcinoma, radiation therapy, hypofractionation, elderly, case report

## Abstract

Merkel cell carcinoma (MCC) is an aggressive cutaneous neuroendocrine neoplasm. Although surgical resection is the first-line treatment for primary MCCs, the characteristics of this disease (i.e., high prevalence in the elderly and in the head and neck region) generate a considerable number of inoperable cases. Radiation therapy shows potential as a definitive treatment alternative to surgery. In definitive radiation therapy for non-resected MCC, 60-66 Gy administered in 2 Gy per fraction and five fractions per week is recommended. However, such frequent visits to the hospital can be a great burden to elderly patients and family members. In this report, we present the case of an 89-year-old patient with MCC (cT2N0M0, stage IIA) of the left cheek. The patient was treated with radiation therapy using a mild hypofractionated schedule (57 Gy provided in 3 Gy per fraction and three fractions per week) targeting the gross tumor. The treatment led to complete tumor remission with well-tolerated acute toxicities at three months post-irradiation. This case indicates that a mild hypofractionated irradiation schedule may achieve tumor control in MCC patients who are inoperable and who cannot make frequent hospital visits.

## Introduction

Merkel cell carcinoma (MCC) is an aggressive cutaneous neuroendocrine neoplasm [1,2]. Surgery (i.e., wide excision) is the standard treatment for primary MCCs [1]. However, MCC is prevalent among the elderly population, in which general anesthesia and surgery carry a high risk [3]. In addition, MCC frequently arises in the head and neck region, where wide excision without functional and cosmetic sequelae is difficult [3]. For these subsets of patients, radiation therapy shows potential as a definitive treatment alternative to surgery because MCCs are highly radiosensitive [4]. A literature review shows that in-field control rates and five-year overall survival rates for MCC treated with definitive radiation therapy are 75%-85% and 40%-60%, respectively [3].

In definitive radiation therapy for non-resected MCC, a common schedule is 60-66 Gy administered in 30-33 fractions and five fractions per week [1]. However, this requires frequent visits to the hospital, which can be a great burden to elderly patients. Here, we report a case of primary MCC of the head and neck region treated with radiation therapy using a mild hypofractionated schedule consisting of 57 Gy administered in 3 Gy per fraction and three fractions per week targeting the gross tumor. The treatment led to complete remission (CR) with well-tolerated acute toxicities. These results support the use of mild hypofractionated radiation therapy schedules for tumor control in elderly and inoperable MCC patients.

## Case presentation

An 89-year-old Japanese woman diagnosed with primary MCC of the left cheek was referred to the department of radiation oncology from the department of dermatology for definitive irradiation in April, 2019. Her performance status was 1 assessed by the Eastern Cooperative Oncology Group definition. She had a history of hypertension and valvular disease.

**Figure 1:**
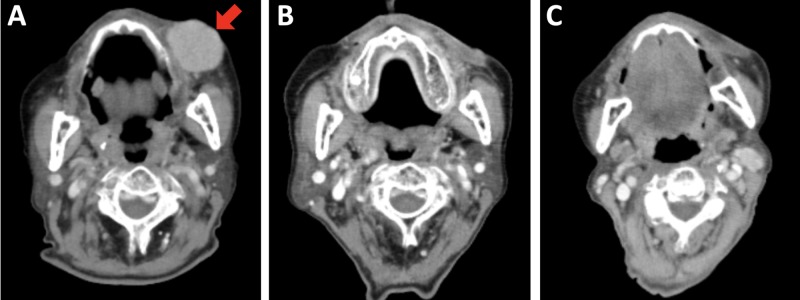
Computed tomography images of Merkel cell carcinoma. (A) Before initiation of radiation therapy. An arrow shows the tumor (36 mm in diameter) (B) In the middle of radiation therapy (i.e., at 36 Gy in 12 fractions). (C) Three months after initiation of radiation therapy.

In January, 2019, the patient noticed a papule on the left cheek. The lesion was diagnosed as a bacterial infection by the dermatologist. Despite treatment with antibiotics, the lesion showed rapid growth and formed a red dome-like tumor measuring 36 mm in diameter in March, 2019. Immunohistochemical analysis of the biopsied specimen showed positivity for CK20 (with a paranuclear dot-like pattern), chromogranin A, synaptophysin, CD56, and cytokeratin AE1/AE3, and negativity for S-100 and HMB-45. Computed tomography (CT) examination showed a spherical tumor mass in the left cheek (Figure 1A) and no lesions indicative of metastases in the regions from the head through the pelvis. The patient was diagnosed with MCC, cT2N0M0, stage IIA [5].

**Figure 2:**
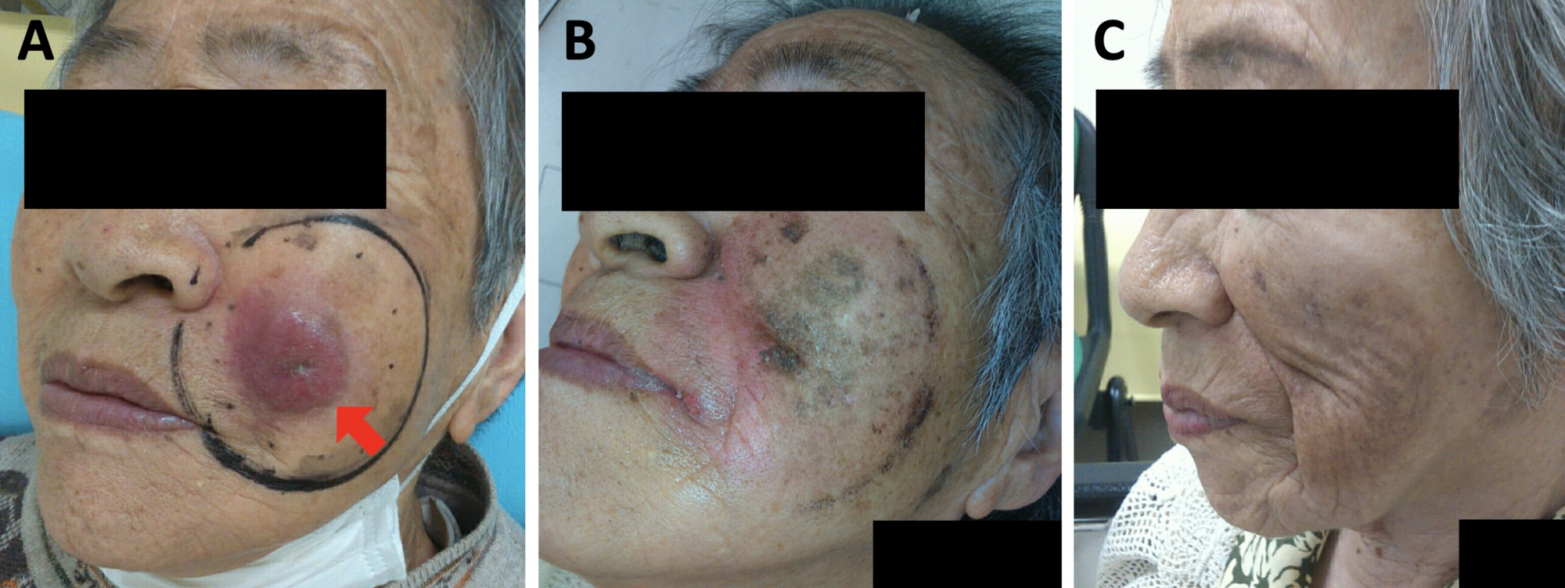
Appearance of the Merkel cell carcinoma. (A) Before initiation of radiation therapy. An arrow shows the tumor and a black circle shows the irradiation field targeting the gross tumor with a margin of 2–3 cm. (B) On the day of completion of radiation therapy. (C) Three months after initiation of radiation therapy.

The patient received radiation therapy with a total dose of 57 Gy administered in 19 fractions (three fractions per week: on Tuesday, Thursday, and Friday). Electrons (12 MeV) were delivered to the gross tumor with a margin of 2-3 cm (Figure 2A) using a 5-mm-thick tissue-equivalent bolus without thermoplastic mask. The energy of the electron beams was kept the same throughout the course with an intention to cover the tumor bed according to tumor shrinkage. The tumor showed rapid remission, and CT images taken at 36 Gy showed near-CR of the tumor (Figure 1B). The tumor became invisible from the skin surface upon completion of radiation therapy (Figure 2B). The treatment was well tolerated; the patient experienced grade 1 dermatitis, grade 1 cheilitis, and grade 1 hyperpigmentation (based on the Common Terminology Criteria for Adverse Effects, version 5.0). No oral mucositis and dysgeusia were observed. At three months after the initiation of radiation therapy, the tumor showed CR (Figure 1C), and the skin surface remained intact (i.e., without defect or ulceration) with mild pigmentation (Figure 2C). CT examinations showed no lymph node or distant metastases were observed in the regions from the head through the pelvis.

After one month, the patient developed two lymph node metastases in the left neck (one in level IA and another in level IB regions). The metastatic nodes and elective nodal regions (i.e., level IA, IB, II, III, IVA, V, VIA, and a part of VII) were treated with additional irradiation (60 Gy and 54 Gy in 30 fractions, respectively) using simultaneous integrated boost intensity-modulated radiation therapy. The treatment led to clinical CR of the irradiated lesion with no evidence of metastasis for another three months. Follow-up by CT every three months up to three years, and every six months thereafter is scheduled to carefully monitor for regional and distant metastases [1].

## Discussion

Here, we present a case of a patient with MCC who selected radiation therapy as an alternative to surgical resection due to advanced age and prevention of cosmetic sequelae. Because frequent hospital visits are a burden for patients and family members, we used a mild hypofractionated schedule (3 Gy per fraction, three fractions per week). In the conventional schedule of five weekly fractions for definitive radiation therapy for non-resected MCC, 60-66 Gy is administered in 30-33 fractions, showing a BED10 (biologically equivalent dose with the a/b ratio of 10) of 72.0-79.2 Gy [1]. Therefore, the total dose was set at 57 Gy in 19 fractions to ensure that the BED10 fell within this range, i.e., 74.1 Gy. As a result, the patient achieved complete tumor remission at least three months post-irradiation with no severe acute toxicities. Data on late toxicities are lacking in this case because of the short follow-up period; however, a meta-analysis of hypofractionated irradiation for skin cancer indicates that the BED3 of 114 Gy calculated for the present case results in good- and fair-cosmetic outcomes in approximately 70% and 15% of patients, respectively, with a median follow-up of 36 months (range, 12-77 months) [6]. Taken together, these data indicate the possibility that the mild hypofractionated schedule is feasible for radiation therapy in patients with primary non-resected MCC. Further investigation is needed to validate the present results because there is little data on the effects of radiation therapy for this rare disease. In addition, the proposed schedule is not feasible for non-MCC malignancies of the head and neck region, in which treatment often fails because of accelerated tumor cell repopulation during long overall treatment times [7].

Prophylactic irradiation for clinically node-negative cases remains controversial because the treatment (i) increases the risk for lymphedema and (ii) can be performed at a later time point as salvage treatment for locoregional recurrence [1,8,9]. In the present case, prophylactic irradiation to the nodal basin was not performed as initial treatment for these reasons after obtaining consent from the patient. In addition, sentinel lymph node biopsy was not performed based on patient's rejection. Although the patient experienced lymph node metastasis in the neck, the disease was salvaged by additional irradiation.

## Conclusions

We present a case of primary MCC of the head and neck treated with definitive radiation therapy using a mild hypofractionated schedule consisting of 57 Gy administered in 3 Gy per fraction and three fractions per week. The treatment led to complete tumor remission with well-tolerated acute toxicities at three months post-irradiation. 

## References

[1] Bichakjian CK, Olencki T, Aasi SZ (2018). Merkel Cell Carcinoma, Version 1, 2018, NCCN Clinical Practice Guidelines in Oncology. J Natl Compr Canc Netw.

[2] Villani A, Fabbrocini G, Costa C, Carmela AM, Scalvenzi M (2019). Merkel cell carcinoma: therapeutic update and emerging therapies. Dermatol Ther.

[3] Gunaratne DA, Howle JR, Veness MJ (2017). Definitive radiotherapy for Merkel cell carcinoma confers clinically meaningful in-field locoregional control: a review and analysis of the literature. J Am Acad Dermatol.

[4] Leonard JH, Ramsay JR, Kearsley JH, Birrell GW (1995). Radiation sensitivity of Merkel cell carcinoma cell lines. Int J Radiat Oncol Biol Phys.

[5] Amin MB, Edge S, Greene F (2017). The American Joint Committee on Cancer: AJCC Cancer Staging Manual.

[6] Zaorsky NG, Lee CT, Zhang E, Keith SW, Galloway TJ (2017). Hypofractionated radiation therapy for basal and squamous cell skin cancer: a meta-analysis. Radiother Oncol.

[7] González Ferreira JA, Jaén Olasolo J, Azinovic I, Jeremic B (2015). Effect of radiotherapy delay in overall treatment time on local control and survival in head and neck cancer: review of the literature. Rep Pract Oncol Radiother.

[8] Sims JR, Grotz TE, Pockaj BA (2018). Sentinel lymph node biopsy in Merkel cell carcinoma: the Mayo Clinic experience of 150 patients. Surg Oncol.

[9] Tseng YD, Parvathaneni U (2018). Primary radiation therapy for Merkel cell carcinoma. Int J Radiat Oncol Biol Phys.

